# Venous congestion from a vascular waterfall perspective: reframing congestion as a dynamic Starling resistor phenomenon

**DOI:** 10.1186/s40635-025-00828-7

**Published:** 2025-11-24

**Authors:** Ricardo Castro, Eduardo Kattan, Jaime Retamal, Glenn Hernández, Michael R. Pinsky

**Affiliations:** 1 Departamento de Medicina Intensiva, Facultad de Medicina, Pontificia Universidad Católica de Chile, Av. Diagonal Paraguay #362 Piso 6, Santiago Centro, 8330049 Santiago, RM Chile; 2 Hospital Clínico UC-CHRISTUS, Pontificia Universidad Católica de Chile, Santiago, Chile; 3 Department of Critical Care Medicine, University of Pittsburgh, Pittsburgh, PA USA

## Abstract

The vascular waterfall phenomenon, rooted in Starling resistor principles, describes how blood flow becomes independent of downstream pressure when intraluminal pressure falls below a critical closing pressure (Pcrit). This review first introduces the classic arterial vascular waterfall, where local Pcrit enables organ-specific autoregulation of blood flow despite varying metabolic demands. Building on this framework, we extend the concept to the venous side, where similar mechanisms govern venous return and protect against congestion. The pulmonary vascular waterfall serves as a prototype, illustrating how alveolar pressures redefine downstream limits, shaping the effects of mechanical ventilation and positive end-expiratory pressure (PEEP). In valveless venous beds such as the hepatic veins, a reverse vascular waterfall may occur when elevated downstream pressure, typically right atrial pressure, causes brief, localized backflow buffered by vessel collapse and the emergence of a new Pcrit. These mechanisms explain organ-specific vulnerabilities to venous congestion: organs with effective venous waterfalls, such as the liver and intestine, can partially buffer overload, whereas the kidney, lacking such protection, is highly susceptible to venous pressure-dependent injury. Clinical implications include refined approaches to PEEP titration, fluid management balancing responsiveness with tolerance, and congestion assessment through Doppler ultrasound. Reframing congestion as a dynamic Starling resistor process explains why similar CVP elevations produce heterogeneous organ effects and provides a mechanistic basis for individualized, physiology-guided critical care.

## Introduction

### Background: revisiting the vascular waterfall concept

Blood flow through the vascular system is primarily driven by pressure gradients. From a physics standpoint, this relationship follows a linear hydraulic model in which blood flow (*Q*) equals the pressure difference between an upstream and a downstream point divided by the segment’s resistance. This principle is analogous to Ohm’s law (*Q* = *ΔP/R*) [1, 2].

**Fig. 1:**
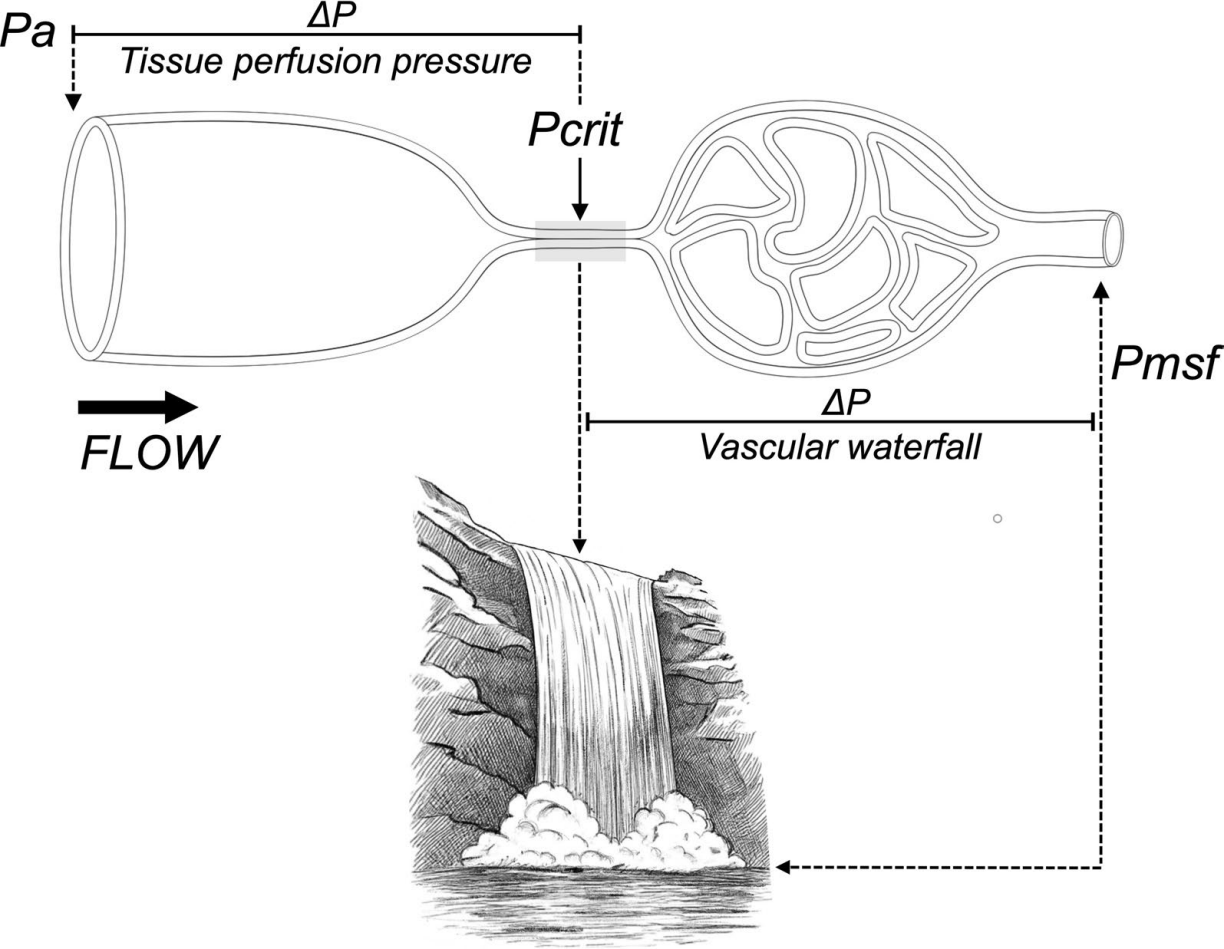
Schematic representation of the vascular waterfall concept. Blood flow from the arterial side (Pa) toward the venous circulation is overall determined by the pressure difference between Pa and venous pressure. Tissue perfusion pressure is determined by the gradient between Pa and the critical closing pressure (Pcrit). When a choke point occurs (Pcrit), forward flow becomes independent of Pa and is governed by the pressure gradient between Pcrit and the mean systemic filling pressure (Pmsf), which defines the vascular waterfall phenomenon and segment. In this model, Pcrit acts as the effective upstream pressure, illustrating that once blood passes over the edge (i.e., Pcrit), its flow is unaffected by further decreases in downstream pressure, highlighting the dissociation between venous pressure and forward flow

The *vascular waterfall* describes a situation in which blood flow becomes independent of downstream pressure once the intraluminal pressure falls below an opposing tissue or vasomotor pressure, known as the *critical closing pressure* (*Pcrit*). When this occurs, the vessel partially collapses, and the flow is primarily governed by upstream driving pressure and vessel wall forces, rather than by the downstream pressure measured at its outlet [3, 4]. Such collapsible segments are known as *Starling resistors*, named after Ernest Starling’s classic experiments [5] (Fig. 1; Table 1).

**Table 1:** Definitions of relevant concepts related to the venous vascular waterfall in technical and lay terms

Concept	Technical definition	In lay terms
Critical closing pressure (Pcrit)	The pressure point below which a vessel collapses and blood flow stops. It marks the threshold that defines a vascular waterfall, separating pressure-dependent from pressure-independent flow	The minimum pressure threshold that keeps a blood vessel propped open against external squeezing forces. Below this point the vessel walls meet and flow stops, like a garden hose collapsing under pressure when someone steps on it
Mean systemic filling pressure (Pmsf)	The effective upstream pressure driving venous return. It represents the average pressure within the systemic circulation when there is no flow and vascular tone remains unchanged	The baseline pressure that exists throughout all blood vessels if the the heart stops, created by blood volume stretching elastic vessel walls. It reflects the stored elastic energy that normally drives blood back toward the heart
Starling resistor	Collapsible segments of conducting tubular structures that limit flow. Determines a dynamic process in which flow through flexible tubes, such as blood vessels or small airways, becomes influenced by external pressure that surpasses the pressure inside the lumen. When surrounding tissue or smooth muscle tone compresses the structure, it partially or completely collapses. This mechanism commonly occurs in normal blood vessels supplying metabolically active tissues and in airways during expiration	A functional structure that behaves like a soft tube squeezed from the outside: when external pressure rises, the tube narrows or closes, controlling flow regardless of the pressure downstream from the pinch point
Vascular waterfall	A physiological phenomenon in which blood flow through a vessel or vascular bed becomes independent of downstream pressure once the intraluminal pressure falls below a critical pressure, due to vessel collapse or external compression. This is modeled after collapsible tube dynamics and the Starling resistor, commonly observed in arterial (for autoregulation) and venous (for buffering congestion) circuits, as well as in pulmonary zones	A flow behavior where blood flows over a pressure ridge or threshold: once it passes that threshold, lowering downstream pressure no longer increases flow, so blood flow becomes independent of what lies below and allows local redistribution of flow downstream the choke point
Reverse waterfall	A descriptive term for brief, phasic backflow of venous blood in valveless vascular beds when downstream pressure transiently exceeds upstream pressure across a Starling resistor segment. This creates a temporary inversion of the pressure gradient, buffered by vessel collapse, preventing sustained system-wide blood flow reversal	A brief backward pulse that occurs when downstream pressure momentarily spikes above upstream pressure. The vessel briefly collapses at the choke point, limiting this backward motion to a fleeting ripple rather than a true reversal of circulation
Venous congestion	A hemodynamic state in which elevated venous pressure is transmitted backward into the smaller vains down into the microcirculation, producing capillary engorgement, edema, and impaired organ function	It is what happens when blood backs up in the veins and leaks into tissues because pressure cannot escape, leading to swelling and organ strain
Buffering capacity	The ability of a vascular bed to absorb increases in venous pressure without passing them to the capillaries	The organ’s “shock absorber” for venous pressure: some, like the liver, cushion it well; others, like the kidney, do not

Classically, the arterial vascular waterfall explains why peripheral blood flow can vary widely across tissues despite a common upstream arterial pressure, reflecting their changing metabolic demand [3, 5–8]. In the waterfall model, the local vascular bed *Pcrit* serves as the effective outflow pressure for forward flow, rising to limit inflow or falling to augment local inflow [6]. This also explains how organ perfusion may be sustained at a level sufficient to support local metabolic function even when arterial pressure decreases, provided that arterial pressure remains above this threshold and a gradient persists [3, 9]. The concept is central to defining the determinants of tissue autoregulation of blood flow, ischemic limits, and reconciling discrepancies between systemic pressure and effective perfusion pressure [10]. Studies across diverse vascular territories confirm the waterfall phenomenon, underscoring its relevance for autoregulation, microvascular flow distribution, interpretation of arterial pressure–flow relationships, and the hemodynamic effects of vasopressors in critical illness [8, 10–14].

Importantly, vascular waterfall phenomena also occur in the systemic venous circuit across various vascular beds, explaining why specific organs, such as the gut, can partially buffer vascular congestion during acute episodes of venous hypertension. In contrast, other organs, like the kidney, cannot. The processes that define venous waterfalls arise either under physiological conditions (when local downstream venous pressures are elevated, as in the gut with its naturally higher portal pressure) or under pathological states (when tissue pressures are abnormally increased, such as with intra-abdominal or intracranial hypertension). While the arterial waterfall has long been established as a cornerstone of autoregulation, its venous counterparts have received less attention. Extending the concept to the venous side highlights how similar mechanisms, though operating under different pressures, critically determine venous return and the susceptibility to upstream organ congestion.

### Transition to venous waterfall perspective

In the venous system, a similar choke point can form. When local vein pressure falls below a certain limit or threshold (*Pcrit*), venous outflow becomes independent of right atrial pressure (*Pra*), creating a venous waterfall [3, 6]. In this condition, the local venular pressure, often close to the mean systemic filling pressure (*Pmsf*), acts as the upstream driving pressure, while *Pcrit* represents the effective downstream limit [15]. In this review, we use *Pra* to describe the right ventricular filling pressure and central venous pressure (*CVP*) as the extrathoracic back pressure equivalent to venous drainage. Under normal conditions, during apnea, *Pra* and *CVP* are similar. Changes in intrathoracic pressure will directly alter *CVP* and may create a vascular waterfall between extrathoracic *CVP* and *Pra*. Commonly, during forced inspiration, especially against an inspiratory airflow resistor (e.g., constricted bronchi in asthma) or if lung parenchymal tissue is stiff (e.g., pulmonary fibrosis, acute respiratory distress syndrome), intrathoracic pressure decreases below atmospheric pressure, causing the large veins’ pressure to also become sub-atmospheric and collapse as they enter the thorax. This limits further increases in venous return that would otherwise occur if this vascular collapse did not happen, even though *CVP* may not decrease as much as *Pra,* depending on where the central venous catheter is placed within the thorax [16].

Similar waterfall phenomena occur whenever tissue pressure around a vein exceeds its intraluminal pressure, such as during intra-abdominal or intracranial hypertension or localized compartment syndromes [17–22]. In that case, the vein partially collapses, creating a local choke point. The result is a smaller pressure gradient for venous return (*Pcrit* to *Pra*) and a higher pressure needed to reopen the vessel. In cases when a higher local *Pcrit* is part of normal physiology, as with portal pressure being higher than *Pra*, transient increases in *Pra* below this local *Pcrit* value will not alter upstream venous pressure nor the rate of local venous return [23]. However, in pathological states, *Pra* can acutely rise above *Pcrit*. Common examples include acute volume overload in the setting of right ventricular dysfunction and/or acute pulmonary hypertension (e.g., hyperinflation, asthma), increased intrathoracic pressure (e.g., tension pneumothorax), or increased pericardial pressure (e.g., cardiac tamponade) [24–27]. In these cases, the newly elevated *Pra* acts as back pressure on venous return, making venous congestion more uniform across organs.

It is essential to remember that different venous beds exhibit varying levels of pressure buffering or protection against acute elevations of *Pra,* depending on their Starling resistor existing mechanisms. If a venous vascular waterfall is present, then increasing *Pra* will not alter local venous pressure or blood drainage until *Pra* exceeds these local *Pcrit* values. Conversely, in vascular territories without a venous waterfall, congestion is transmitted directly back to the capillary network, favoring edema and dysfunction. This distinction between organs protected by venous waterfalls and those that are not forms the conceptual core of this review.

## The venous vascular waterfall

### Conceptual framework

Arterial and venous vascular waterfalls share similar underlying mechanisms, but their physiological and clinical implications differ markedly. On the arterial side, waterfalls enable organ-specific autoregulation, matching blood flow to metabolic demand while protecting tissues from hypoperfusion during transient reductions in arterial inflow pressure up to a critical threshold. In contrast, the venous counterpart, while less clearly defined, serves dual roles: under normal physiological conditions, it dynamically limits venous return to prevent overload (e.g., during forced inspiration or regional pressure changes, as seen in the collapse of thoracic veins or abdominal compartments), and in pathological states, it buffers venous congestion to shape organ-specific vulnerability.

Fessler et al. described a functional vascular waterfall phenomenon in large veins under circumstances such as positive end-expiratory pressure or body positioning [28]. In these common conditions during positive pressure ventilation, intrathoracic pressure can exceed *Pra* during inspiration, limiting global venous return [16]. These observations underscore the reality that venous return is not uniformly governed by a simple *Pmsf* minus *Pra* pressure gradient, but instead by regionally variable resistors that may emerge or resolve depending on physiological demands or pathological states. Although *Pmsf*, *Pcrit*, and the vascular waterfall are theoretical constructs, practical bedside methods exist to approximate them in clinical settings (Table 2). These methods highlight that the vascular waterfall is not merely conceptual, but observable at the bedside, aiding in real-time hemodynamic optimization.

**Table 2:** Bedside and computational methods to estimate Pcrit and Pmsf with clinical application

Parameter	Principle/technique	Method description	Clinical context or application	Key references
Pcrit (critical closing pressure)	Zero-flow extrapolation	Plots mean arterial pressure (MAP) against cardiac output (CO) during controlled hemodynamic changes (e.g., fluid challenge); the extrapolated intercept at zero CO represents Pcrit	Estimates effective perfusion pressure (MAP–Pcrit); applicable in controlled or research settings	[10, 15, 105]
	Inspiratory hold maneuvers	Stepwise end-inspiratory holds (5–35 cmH₂O) in ventilated patients alter CO and MAP; Pcrit is extrapolated from MAP–CO regression	Bedside estimation during mechanical ventilation; allows dynamic assessment of the arterial waterfall	[8, 15, 106]
	Arm stop-flow technique	Cuff occlusion stops arterial flow; subsequent arterial–venous pressure equilibration defines analogue Pcrit	Non-ventilated or spontaneously breathing patients; noninvasive surrogate of Pcrit	[107]
	Automated continuous estimation	Derives tissue perfusion pressure (TPP = MAP–Pcrit) via waveform analysis; validated in ICU cohorts	Continuous TPP monitoring improves risk prediction beyond MAP or SVR	[10]
	Dynamic arterial elastance (Eadyn)	Relates pulse pressure variation (PPV) to stroke volume variation (SVV); changes in vascular tone reflect shifts in Pcrit	Evaluates vasopressor (e.g., norepinephrine) responsiveness; links tone modulation to perfusion	[14, 108]
	Pressure decay or waveform analysis	Fits arterial pressure decay during diastole or arrest to compartmental or pulse contour models for real-time Pcrit estimation	Research or high-fidelity monitoring applications	[109–111]
Pmsf (mean systemic filling pressure)	Venous return curve (zero-flow method)	Derived from transient flow cessation (e.g., circulatory arrest or stop-flow experiments); Pmsf corresponds to equilibrated mean pressure	Classical research approach validating Guyton’s model	[1, 10]
	Inspiratory hold maneuvers	Temporary increases in intrathoracic pressure shift venous return; extrapolation of zero-flow pressure provides bedside Pmsf estimate	Mechanically ventilated patients; practical surrogate for stressed blood volume	[15, 112]
	Arithmetic/computational analog (Pmsa)	Calculated from CO, MAP, and right atrial pressure (RAP), adjusted for patient characteristics (age, height, weight). Implemented via *iGuyton©* app	Bedside estimation of “effective circulatory blood volume”; Pmsa usually within ± 2 mmHg of Pmsf	[113]
	Gradient approach (Pcrit–Pmsf)	The pressure difference between Pcrit and Pmsf defines the *functional vascular waterfall*, representing the effective driving pressure for flow	Integrates arterial and venous components of the Starling resistor model	[15]

*MAP* mean arterial pressure, *CO* cardiac output, *Pcrit* critical closing pressure, *Pmsf* mean systemic filling pressure, *TPP* tissue perfusion pressure, *SVR* systemic vascular resistance, *Pmsa* mean systemic filling pressure analog, *Eadyn* dynamic arterial elastance, *PPV* pulse pressure variation, *SVV* stroke volume variation

**Table 3:** Organ-specific venous waterfall protection and vulnerability to congestion

Organ	VW present	Buffering capacity	Primary mechanisms	Congestion vulnerability	Clinical manifestations	Monitoring
Kidney	No	Low	None effective	High	AKI, oliguria, ↑Cr	RVR index, VExUS
Liver	Partial	Intermediate	Sinusoidal resistance, HABR, compliance	Moderate	↑transaminases, ↑bilirubin	HV Doppler, portal pulsatility
Intestine	Partial	Intermediate	Portal compliance, sympathetic tone	Moderate-High	Ileus, barrier dysfunction, IAH	SMV Doppler, ICP

*VW* vascular waterfall, *HABR* hepatic arterial buffer response, *AKI* acute kidney injury, *Cr* creatinin, *HV* hepatic vein, *RVR* renal venous resistive, *SMV* superior mesenteric vein, *IAH* intra-abdominal hypertension

See main text for details

In normal steady states, venous return depends on the pressure difference between *Pmsf* and *Pra. Pmsf* defines the upstream driving pressure for the circuit, and *Pra* represents the downstream limit. The conditions for segmental collapse determine whether venous flow is pressure dependent or pressure independent [29]. From its inception, *Pmsf* has been regarded as a *lumped parameter* representing all vascular beds. Because local venous pressures are generally low, inter-organ differences can often be approximated by *Pmsf*. However, this simplification overlooks critical heterogeneity: some organs are especially vulnerable to dysfunction from acute venous hypertension, whereas others are relatively protected by local venous waterfall mechanisms. This perspective does not overturn the classical view of veins as passive capacitance vessels but refines it, acknowledging that downstream resistors can locally modulate venous drainage.

From a clinical perspective, understanding venous flow under the waterfall framework helps clinicians interpret congestion beyond absolute *CVP* values. Once a local *Pcrit* develops, venous outflow becomes temporarily independent of *Pra*, so an increased *CVP* does not necessarily worsen drainage until a threshold is surpassed. Each vascular bed possesses its own choke point, determined by surrounding tissue pressure and vessel tone, making congestion a spectrum of regional phenomena rather than a uniform process. This perspective explains why similar elevations in *CVP* can produce very different patterns of organ dysfunction within the same patient and emphasizes the importance of assessing congestion through regional pressure gradients and thresholds rather than relying solely on *CVP* measurements.

### Pulmonary vascular waterfall as a prototype

**Fig. 2:**
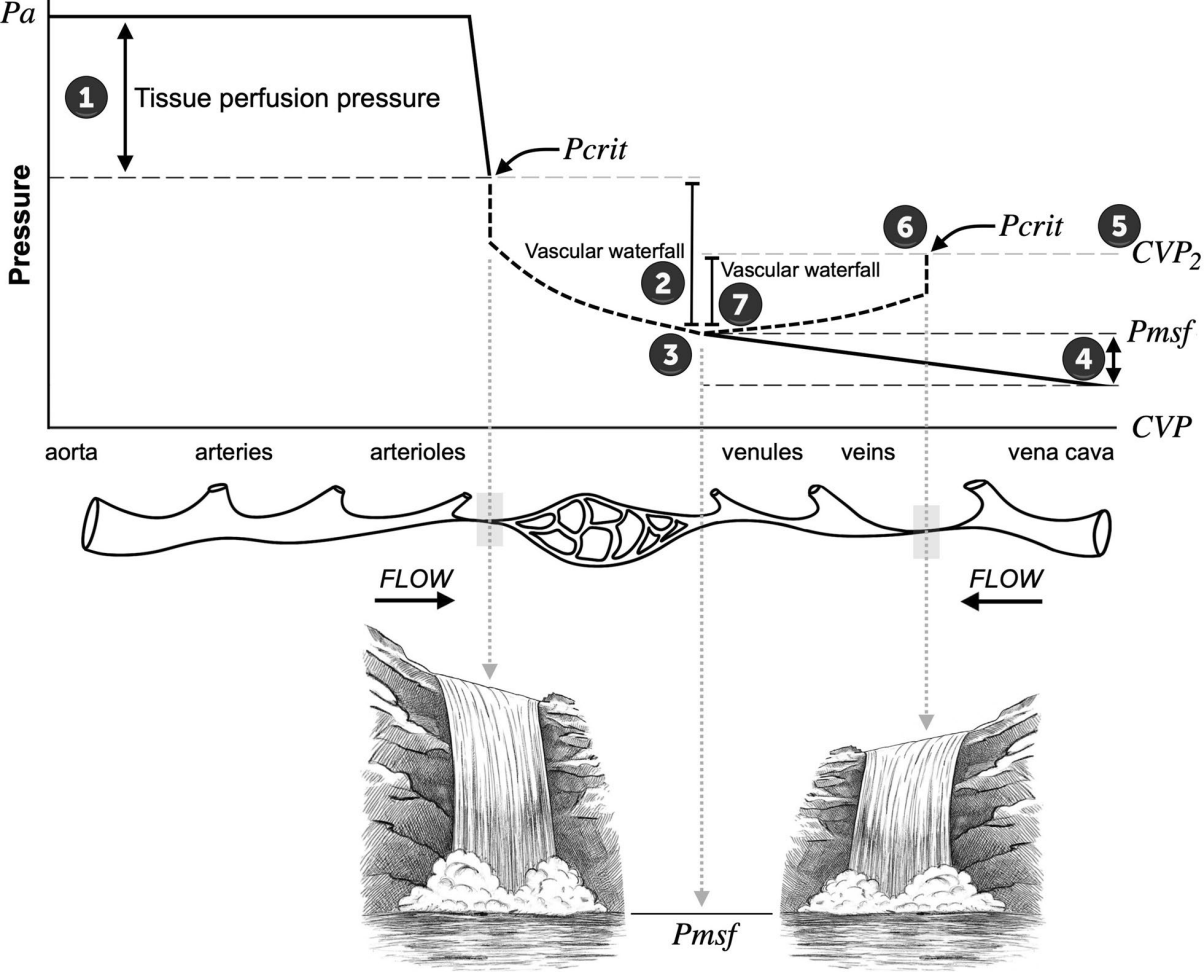
Comprehensive circulatory model integrating critical closing pressures and vascular waterfall phenomena. (1) Tissue perfusion pressure is defined by the difference between mean arterial pressure (*Pa*) and the arterial critical closing pressure (*Pcrit*). (2) The arterial vascular waterfall occurs when flow becomes independent of downstream pressure once *Pa* exceeds *Pcrit*. (3) The mean systemic filling pressure (*Pmsf*) normally acts as the effective downstream pressure for the arterial waterfall, while simultaneously serving as the upstream pressure for venous return when central venous pressure (*CVP*) is lower. (4) Under physiological conditions, venous return is governed by the pressure gradient between *Pmsf* and *CVP*. (5) An abnormally elevated central venous pressure (*CVP*_*2*_) becomes the upstream pressure of the system, transmitting backward pressure into the venous circulation, thereby impairing venous return. (6) The emergence of a venous critical closing pressure (venous *Pcrit*) partitions the circuit into two independent pressure domains. (7) This venous *Pcrit* buffers the backward transmission of *CVP*_*2*_ (dam effect) and, relative to *Pmsf*, creates a local “reverse vascular waterfall” that generates phasic backward venous flow at a lower pressure than would occur in the absence of such a choke point. (8) Overall, if *CVP* approaches/exceeds *Pmsf*, global venous return plateaus via great-vein collapse; any reverse flow is limited to valveless beds and is transient

The lung offers the most distinct and well-studied example of vascular waterfall behavior. First described by West in 1964 to explain the vertical distribution of pulmonary blood flow, it remains the most experimentally validated expression of this principle [29]. In this model, alveolar and pleural pressures redefine the effective downstream pressure, creating conditions in which pulmonary flow can become intermittently independent of venous pressure (Fig. 2).

Because pulmonary arterial pressure is relatively low, hydrostatic forces make it lower in the upper lung regions and higher in the lower regions. In an upright patient of average height and at rest, the hydrostatic pressure drops in the pulmonary arteries as they ascend in the chest and usually exceeds pulmonary artery pressure, and pulmonary flow to those apical regions ceases because pulmonary artery pressure is zero. Their alveolar PO_2_ remains high, and their alveolar PCO_2_ remains low. These areas of the lung are referred to as Zone 1 regions and are explained by gravity-induced hydrostatic forces. Beneath them lie Zone 2 areas, where hydrostatic pressure also exceeds pulmonary venous pressure, even though pulmonary artery pressure can still create flow. Thus, alveolar flow depends only on the gradient between pulmonary artery and alveolar pressures, independent of pulmonary venous pressure; this West zone 2 phenomenon defines the pulmonary vascular waterfall. Further down the lung, flow increases from the start of Zone 2 until pulmonary venous pressure exceeds alveolar pressure and thus becomes the effective back pressure to pulmonary blood flow, defining Zone 3 conditions. In this region, both pulmonary arterial and venous pressures increase in parallel as one moves downward, so the pressure gradient driving flow remains constant [29–31].

Since its early description in 1938 [32], positive end-expiratory pressure (PEEP) has become a cornerstone of modern mechanical ventilation. In ventilated patients or in obstructive lung disease with expiratory flow limitation, end-expiratory alveolar pressure can exceed atmospheric pressure. With applied PEEP, alveolar pressure equals the set value; in hyperinflated lungs with auto-PEEP, alveolar pressure rises heterogeneously. Sustained elevation of intrathoracic pressure by either mechanism can influence the occurrence of venous waterfalls in the systemic circulation [33]. By consistently raising intrathoracic pressure, PEEP may determine whether venous waterfalls manifest in the systemic venous circulation.

PEEP prevents alveolar collapse, reduces shunt, and limits cyclic opening–closing of unstable units [28, 29, 32, 34, 35], but it can also impair venous return and CO depending on volume status, intrathoracic pressure, and lung recruitability [36–39]. From a venous waterfall perspective, this impairment reflects the rise in transpulmonary pressure [29]. By increasing end-expiratory lung volume, PEEP elevates intrathoracic pressure and thereby artifactually raises *CVP*, the external correlate of *Pra*. This apparent rise in *CVP* reduces the pressure gradient for venous return, even when *Pmsf* is unchanged or increased, thus accounting for the fall in CO during positive -pressure ventilation [35, 40]. Moreover, the hemodynamic impact of PEEP is also strongly volume dependent. In hypovolemia, high PEEP can collapse the inferior vena cava (IVC) [35] and create a local “reverse waterfall” effect, further restricting venous return. Even with modern protective ventilation strategies that use lower driving pressures, these effects are only attenuated but remain present [37].

At the bedside, the pulmonary circulation illustrates how external pressures redefine vascular behavior. When alveolar or pleural pressures exceed venous pressure, pulmonary flow becomes independent of downstream pressure, manifesting a Starling resistor condition. PEEP and lung inflation can reproduce this same mechanism systemically, creating functional choke points that limit venous return. For the clinician, this means that every adjustment in airway pressure alters not only gas exchange, but also venous drainage from systemic organs. Recognizing these interactions helps anticipate falls in CO or the onset of venous congestion during ventilator management and underscores the need to interpret *CVP* and preload indices while accounting for intrathoracic pressure changes. The following sections explore how these principles apply to the liver, intestine, and kidneys, highlighting organ-specific mechanisms that confer vulnerability or resilience to congestion.

### The reverse waterfall

In this review, the term *reverse waterfall* refers to short, localized episodes of backward venous flow that appear when downstream pressure (*Pra* or an extravascular pressure) briefly exceeds upstream pressure. These events occur mainly in valveless veins such as hepatic or portal territories, and are transient rather than sustained. According to the Guyton framework, venous return becomes negative when Pra rises above Pmsf; in vivo, however, collapse of the great veins at the thoracic inlet limits flow to near zero, preventing global backflow [41, 42]. Thus, any reverse flow is expected to be phasic and regional rather than a true reversal of circulation.

Two contexts support this bounded interpretation. First, respiratory or ventilator-induced *Pra* swings (e.g., high PEEP, forced inspiration, or intra-abdominal hypertension) can transiently invert the local pressure gradient, with venous collapse and zone-like behavior limiting return, phenomena that are breath synchronous, not new equilibria [16, 17, 28]. Second, in valveless beds contiguous with the right atrium, particularly the hepatic veins, Doppler recordings may show systolic flow reversal during right ventricular overload or tricuspid regurgitation, and portal pulsatility can increase under high PEEP. These are genuine but time-limited expressions of local Starling resistor dynamics, not evidence of a sustained reverse circulation [43, 44]. Accordingly, we retain the term *reverse waterfall* in cursive and restrict its use to transient, territory-specific backflow patterns (e.g., hepatic systolic reversal or portal pulsatility spikes) rather than a steady-state hemodynamic condition.

### Organ-specific perspectives (Fig. 2, Fig. 3, and Fig. 4; Table 3)

**Fig. 3:**
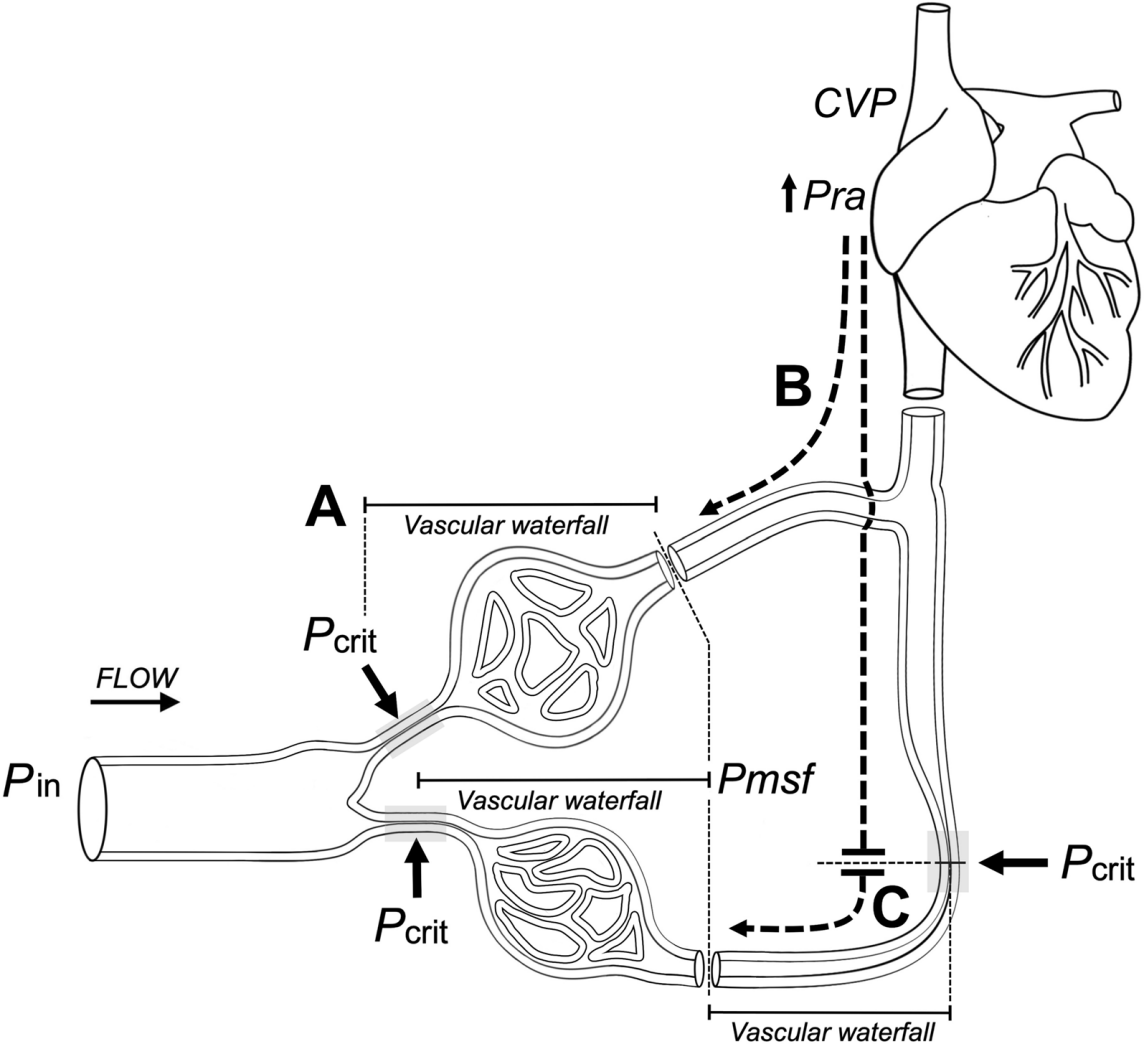
Venous vascular waterfall physiology under normal and congested states. A *Classic vascular waterfall.* In collapsible arteriolar segments, flow depends on the pressure difference between the critical closing pressure (*Pcrit*) and the mean systemic filling pressure (*Pmsf*). Once downstream pressure (*Pmsf*) falls below, flow becomes independent of *Pra*, resembling a forward Starling resistor. B *Congestion without venous waterfall protection.* In organs lacking a venous waterfall (e.g., the kidney), central venous pressure (*CVP*) is directly transmitted backward to the microcirculation. Here, *Pmsf* functions as the effective downstream pressure, abolishing the protective dissociation from *Pra*. This favors renal venous congestion and impaired filtration. C *Congestion with partial venous waterfall protection.* In organs with venous waterfall mechanisms (e.g., liver, intestine), backward transmission of elevated *CVP* is buffered. The new upstream pressure becomes lower than *CVP*, such that only part of the rise in *Pra* is transmitted to the microcirculation. In these territories, multiple vascular waterfalls (e.g., hepatic sinusoids, suprahepatic veins) provide graded protection against venous congestion

**Fig. 4:**
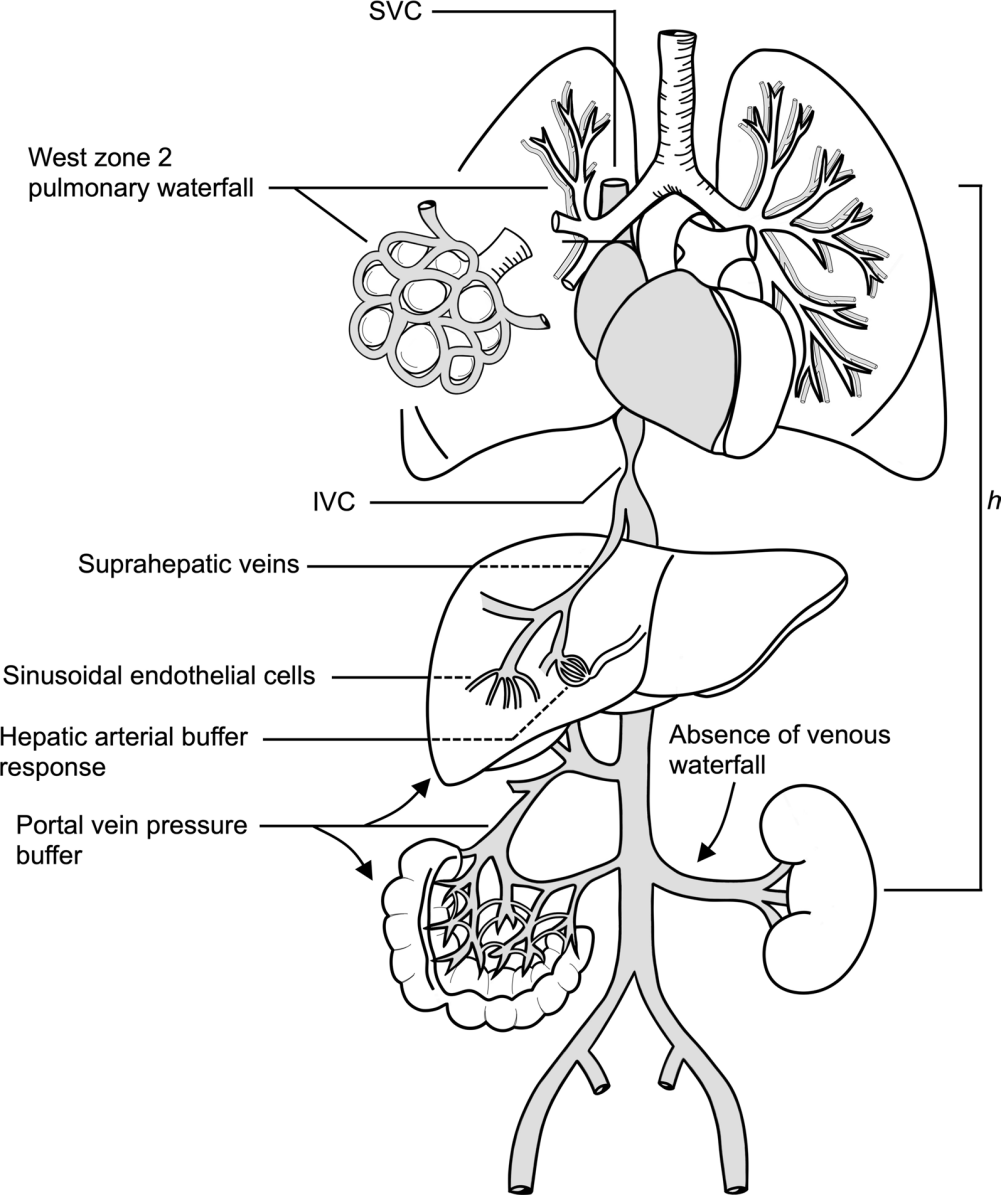
Venous waterfall physiology and protective mechanisms for organ congestion. A *General principle*: venous outflow becomes independent of central venous pressure (*CVP*) once downstream pressure falls below a critical closing pressure (*Pcrit*), with mean systemic filling pressure (*Pmsf*) as the upstream driver. B *PEEP* raises intrathoracic pressure, establishing a new downstream resistor and potentially limiting venous return. C *Liver*: sinusoidal buffering offers partial protection, though high PEEP can propagate portal congestion. D *Kidney*: absence of venous waterfall buffering renders the kidney highly susceptible to pressure-dependent congestion and impaired filtration. E *Intestine*: partial waterfall protection exists, but thin-walled veins are vulnerable to increases in intrathoracic or intra-abdominal pressure. *SVC* superior vena cava, *IVC* inferior vena cava, *h* height

When a venous vascular waterfall is present, it acts as a pressure buffer between the organ and the central veins. Venous systems with strong waterfall behavior are protected from rises in downstream pressure (e.g., *CVP*) until that pressure exceeds their local closing pressure. This is why some organs can maintain venous drainage even when central pressures rise modestly, but this buffering capacity varies widely among vascular territories. The following sections examine how these differences determine some organ responses to congestion, beginning with the liver.

- **Liver.** In animal models, zero-flow sinusoidal pressure reflects a closing pressure, likely mediated by sinusoidal endothelial or Kupffer cells that bulge into the lumen as an active protective mechanism against pressure overload [45–47]. Because sinusoidal filtration is highly pressure sensitive in the absence of a robust oncotic gradient [48], the liver can buffer fluctuations in *CVP* [47]. Protective mechanisms include a hepatosplanchnic waterfall effect whereby IVC flow may recover faster than the superior vena cava (SVC) flow during inspiratory holds, preserving venous return [26]. Additional defenses rely on hepatic vascular compliance and the hepatic arterial buffer response [23, 49]. When hepatic venous pressure remains below a critical threshold set by tissue or sinusoidal pressure, outflow is maintained; once IVC pressure exceeds this level, venous congestion develops [49, 50]. With elevated intrathoracic pressures, transient disequilibrium between hepatic and portal pressures may allow time for compliance and waterfall mechanisms to sustain venous return. However, defenses fail in fluid overload when high *Pmsf* and *Pra* transmit backward pressure more directly [26, 49]. Thus, unlike the kidney, in fluid overload, the liver retains partial protection through its venous waterfall and buffering responses. Still, unlike the intestines, described below, acute and sustained hepatic venous congestion can occur in cardiogenic and obstructive shock states and often results in acute liver injury [51, 52].
- **Intestine.** The intestinal vasculature supports absorption via fenestrated villous capillaries, renewal via crypt stem cell niches, and immune trafficking [53–55], while the dense villous network is highly vulnerable to oxygen fluctuations [56, 57]. Venous congestion is particularly harmful: occlusion leads to early, extensive injury from metabolite accumulation and impaired clearance, with reperfusion triggering "congestion–reperfusion" injury and barrier failure [56–58]. Sympathetic regulation, nitric oxide-mediated vasodilation [59], and portal compliance offer temporary protection to venous congestion [26]. In this scenario, IVC closure may create a local “reverse waterfall” in which *Pmsf* becomes the upstream pressure [37]; together with hepatic outflow resistors [26, 47], this partially buffers backward transmission of systemic venous hypertension. These mechanisms, although incomplete, frame the gut's intermediate and fragile tolerance to venous congestion. When protective mechanisms fail, intestinal edema can increase intra-abdominal pressure and, along with additional contributors such as fluid overload, systemic inflammation, or sepsis, drive the progression to intra-abdominal hypertension (IAH) and ultimately abdominal compartment syndrome [60]. These processes limit intestinal venous outflow independent of *CVP*.
- **Kidney.** The kidney lacks an effective venous waterfall; renal veins transmit elevations in IVC pressure directly to the microcirculation, making glomerular filtration highly sensitive to changes in downstream pressure [61]. Elevated renal venous pressure increases Bowman's capsule hydrostatic pressure, reduces the filtration gradient, and promotes tubular back leak [62]. In fact, even modest venous pressure rises reduce urine output and renal cortical oxygen delivery, as oxygen consumption remains constrained by tubular transport demands [63]. Congestive nephropathy demonstrates this vulnerability: renal dysfunction occurs despite preserved renal arterial pressure due to venous hypertension and interstitial edema compressing peritubular capillaries [61, 64, 65]. Chronic IVC constriction models show progressive renal injury despite preserved cardiac function [39], reflecting low compliance and absence of a renal venous waterfall or a robust arterial buffer mechanism. Hence, the kidney is disproportionately susceptible during fluid overload or elevated *CVP* [39, 49].

In practical terms, understanding venous flow through the vascular waterfall framework helps clinicians interpret congestion beyond absolute *CVP* values. Once a local *Pcrit* develops, venous outflow becomes temporarily independent of *Pra*, so increasing *CVP* does not necessarily worsen drainage until that local threshold is exceeded. Each vascular bed possesses its own choke point, determined by surrounding tissue pressure and vascular tone, making congestion a spectrum of regional phenomena rather than a uniform process. This view explains why similar elevations in *CVP* can produce very different patterns of organ dysfunction within the same patient and emphasizes the importance of assessing congestion through regional pressure gradients and thresholds rather than relying solely on *CVP* measurements.

## Clinical implications

The vascular waterfall perspective emphasizes that congestion is not a uniform state, but the result of complex interactions between upstream venous return, downstream closing pressures, and territory-specific vulnerability. As mentioned, venous hypertension impacts renal function first, hepatic function once hepatic buffer responses are exhausted, and intestinal function last. For clinicians, this physiological principle has direct implications for ventilator management, fluid therapy, and monitoring strategies.

The following sections highlight three central domains, namely PEEP titration, fluid administration, and congestion assessment, where appreciation of venous waterfall physiology can guide individualized patient care.

### PEEP titration

By increasing lung volume, PEEP also raises intrathoracic pressure. When this pressure exceeds *Pra*, it acts as a new resistor to venous return, rendering flow independent of *Pra*. This often reduces CO and worsens venous congestion in susceptible vascular beds, although protective ventilation strategies that limit end-expiratory lung volume mitigate the effect [28, 29, 32, 35–40]. Intravascular volume status modifies the response. Hypovolemia amplifies IVC collapse and phasic *reverse waterfall* behavior [35], while moderate PEEP in hypervolemia can buffer backward venous hypertension and facilitate splanchnic outflow [39].

At the hepatic level, even modest increases in PEEP reduce blood flow and CO without altering MAP [36], while higher levels further impair oxygen delivery, lower venous saturation, and elevate portal pressures, contributing to hepatomegaly, biochemical dysfunction, and worsening hepatic injury with recruitment maneuvers [38, 66, 67]. These changes also compromise intestinal perfusion through CO reduction, reflex vasoconstriction, and elevated portal pressure, with lymphatic outflow impairment fostering both hepatic and intestinal edema and inflammation [67–70]. Renal circulation is even more vulnerable. PEEP decreases renal blood flow mainly via CO reduction, while rising venous pressures are transmitted backward to the microcirculation, impairing glomerular filtration. Reflex increases in arterial tone may preserve arterial pressure despite a decreased CO. Still, clinically and experimentally, higher PEEP consistently correlates with oliguria, sodium retention, and biochemical evidence of renal dysfunction [71–74].

The practical implication is that PEEP should be titrated to the lowest level that secures oxygenation and alveolar stability, with careful attention to venous congestion surrogates, since PEEP may elevate downstream pressures and restrict organ venous outflow and venous return. Intravascular volume status, hepatic portal pulsatility, renal output, biomarkers, mesenteric perfusion risk, and other surrogates warrant consideration when escalating PEEP, particularly in ARDS or when high driving pressures are required in mechanical ventilation [36–40, 66–74].

### Fluid administration

Fluid resuscitation in septic shock aims to restore hypoperfusion by increasing venous return and CO, but the benefit depends on adequacy, timing, and avoidance of fluid overload [75–79]. Fluid responsiveness refers to the heart's ability to increase stroke volume in response to a fluid challenge and is a practical clinical tool for predicting the outcome of fluid administration [80]. Fluid responsiveness is a clinical concept and dynamic measure that serves the purpose of guiding the eventual administration of fluids. It indicates whether a patient will increase CO and, ultimately, tissue perfusion, thus benefiting from additional fluid administration. It is assessed through dynamic tests, such as the passive leg-raising maneuver, fluid challenges, pulse pressure analysis, and stroke volume variations [80–85].

Although dynamic predictors identify fluid responsiveness in only about half of patients early in shock, and up to one-quarter become unresponsive after initial loading [81, 86–88], responsiveness alone does not capture the risk of harm. Because the effects of fluids on CO are transient [89], repeated assessments remain essential [81].

From a venous waterfall perspective, the key limitation of a fluid-first approach is that fluid responsiveness does not equal fluid tolerance [90, 91]. Even when CO rises, organs lacking venous waterfall protection, like the kidney, are exposed to direct transmission of elevated IVC pressure, leading to renal venous hypertension and impaired renal function. Conversely, territories with partial buffering may tolerate modest volume expansion, but eventually succumb to congestion once protective mechanisms are overwhelmed. This heterogeneity explains why fluid accumulation carries disproportionate renal risk despite apparently preserved systemic hemodynamics.

Accordingly, fluid management must integrate congestion assessment alongside fluid responsiveness. Organ-specific surrogates (e.g., portal vein pulsatility, hepatic venous Doppler, renal venous flow indices) provide a hydraulic window into whether local waterfalls buffer or transmit elevated pressures [92]. The heterogeneity of venous waterfall protection implies that "fluid tolerance" varies by organ, and even modest volume increments may precipitate injury when such buffering is absent [23, 43, 49, 50, 93–100]. Recognizing that the role of fluid therapy depends on context, such as acute resuscitation versus stabilization or de-escalation [101, 102], fluid administration may at times function as a tool to augment flow and, at other times, as a determinant of venous congestion. This contextual transition underscores the need for individualized fluid management strategies that balance benefits and risks.

### Congestion assessment

Increasing venous blood volume does not necessarily raise venous pressure if the added volume remains within the unstressed range. However, congestion is not simply a matter of an elevated *CVP*; it reflects how local pressures interact with venous return, regional critical closing pressures, and the intrinsic vulnerability of each organ. The venous waterfall framework provides a mechanistic explanation for why the kidney is disproportionately affected, the gut is intermediate, and the liver is relatively buffered during fluid overload, yet intolerant to abrupt rises in *CVP*. This nuanced physiology supports an individualized assessment of congestion, integrating hemodynamic tests with organ-specific ultrasound markers [92] to guide decisions on when to administer, withhold, or actively remove fluids.

When a local venous closing pressure (*Pcrit*) forms, it can temporarily block the transmission of backward pressure. This may appear as brief flow reversals on Doppler [43]. These findings may indicate the presence of a dynamic local reverse waterfall and serve as a warning signal that local buffering mechanisms are being exceeded. They arise when the interaction between an upstream closing pressure and the outflow pressure in the drainage vein permits oscillation across the choke point. By contrast, in the kidney, which lacks such a mechanism, congestion is transmitted directly to the microcirculation, producing parenchymal injury without a clear reverse-flow ultrasound signature. This distinction likely explains why venous excess ultrasound (VExUS) grades ≥ 2 correlate with AKI in cardiac surgery, cardiorenal syndrome, and general ICU cohorts, but show inconsistent predictive value in sepsis [103, 104], where vascular tone, heterogeneity, and timing of assessment confound interpretation.

Effectively, ultrasound-based analysis of venous Doppler patterns provides a noninvasive window into regional vascular waterfall dynamics. Hepatic vein Doppler demonstrates characteristic systolic flow reversals when *CVP* exceeds local venous critical closing pressure, particularly in the presence of right ventricular dysfunction or tricuspid regurgitation. Portal vein pulsatility increases as elevated *CVP* is transmitted backward through the hepatic circulation, overwhelming local buffering mechanisms [43, 67, 92]. These Doppler changes can serve as real-time markers of the threshold at which venous waterfall protection fails. Similarly, renal venous Doppler can reveal direct transmission of *CVP* elevations to the kidney, where absent buffering mechanisms produce monophasic, continuous flow patterns even with modest congestion [98–100]. Integrating these organ-specific Doppler findings with conventional VExUS grading provides a more granular assessment of regional congestion and helps identify which organs are most at risk from further volume loading or should be prioritized during decongestive interventions.

## Conclusion

The vascular waterfall paradigm, long recognized in the arterial domain, is highly relevant on the venous side, where congestion often dictates outcomes in critical illness. The presence, absence, or partial effectiveness of venous waterfall mechanisms determines organ-specific susceptibility: the liver retains partial buffering, the kidney lacks protective capacity, and the intestine represents an intermediate case. By recognizing that both venous and extravascular pressures can establish effective downstream resistors, congestion is reframed as an active physiological process mediated by Starling resistor dynamics, rather than a passive consequence of an elevated *CVP*. This perspective provides a mechanistic explanation for the dissociation between venous pressures and organ injury and must inform individualized strategies for fluid resuscitation, ventilatory support, and decongestive therapy. Future research should aim to integrate noninvasive vascular waterfall assessment into multimodal monitoring and to develop therapeutic strategies that explicitly account for venous waterfall physiology.

## Take-home message

The venous vascular waterfall framework explains why central venous pressure does not uniformly translate into organ congestion. Recognizing organ-specific buffering and critical closing pressures offers a new physiological basis for individualized fluid and ventilatory management.

## Clinical takeaways

1. The venous vascular waterfall appears when venous outflow becomes independent of downstream pressure due to vessel collapse at a critical closing point. This explains why similar central venous pressure (*CVP*) values can affect organs differently and why venous return must be evaluated via pressure gradients and thresholds, not absolute values.
2. When a venous vascular waterfall is present, it acts as a pressure buffer between the organ and the central veins. Rising *CVP* does not immediately increase local venous pressure because the waterfall maintains a threshold, the critical closing pressure, below which flow remains stable.
3. Congestion should be assessed in terms of pressure gradients rather than absolute numbers, because congestion develops when local pressures surpass the organ-specific closing threshold, not simply when *CVP* is elevated.
4. Every change in airway pressure influences venous return. Adjustments in PEEP that improve oxygenation may simultaneously reduce venous drainage and cardiac output.
5. Clinicians should recognize that the kidney is particularly vulnerable to venous hypertension. Even modest elevations in pressure can impair renal function before other organs exhibit signs of congestion.
6. In practice, fluid administration, ventilatory adjustments, and vasoactive support must be considered as interdependent modulators of pressure gradients. Each intervention can alter the venous waterfall balance, influencing organ perfusion and congestion.

## Data Availability

Not applicable. This article does not include original datasets; all information is derived from previously published sources cited in the reference list.

## Abbreviations

AKI: Acute kidney injury
ARDS: Acute respiratory distress syndrome
CO: Cardiac output
*CVP*: Central venous pressure
MAP: Mean arterial pressure
Pa: Arterial pressure
PEEP: Positive end-expiratory pressure
*Pcrit*: Critical closing pressure
*Pmsf * (or Pms): Mean systemic filling pressure
*Pra*: Right atrial pressure
TPP: Tissue perfusion pressure
VW: Vascular waterfall
VExUS: Venous excess ultrasound score
